# Detection and Differentiation of Multiple Viral RNAs Using Branched DNA FISH Coupled to Confocal Microscopy and Flow Cytometry

**DOI:** 10.21769/BioProtoc.3058

**Published:** 2018-10-20

**Authors:** Nicholas van Buuren, Karla Kirkegaard

**Affiliations:** Department of Genetics, Stanford University School of Medicine, Stanford, CA, 94305, USA

**Keywords:** RNA flow cytometry, RNA FISH, Branched DNAs, HCV, Drug Resistance, Genetic selection, Viral evolution

## Abstract

Due to the exceptionally high mutation rates of RNA-dependent RNA polymerases, infectious RNA viruses generate extensive sequence diversity, leading to some of the lowest barriers to the development of antiviral drug resistance in the microbial world. We have previously discovered that higher barriers to the development of drug resistance can be achieved through dominant suppression of drug-resistant viruses by their drug-susceptible parents. We have explored the existence of dominant drug targets in poliovirus, dengue virus and hepatitis C virus (HCV). The low replication capacity of HCV required the development of novel strategies for identifying cells co-infected with drug-susceptible and drug-resistant strains. To monitor co-infected cell populations, we generated codon-altered versions of the JFH1 strain of HCV. Then, we could differentiate the codon-altered and wild-type strains using a novel type of RNA fluorescent *in situ* hybridization (FISH) coupled with flow cytometry or confocal microscopy. Both of these techniques can be used in conjunction with standard antibody-protein detection methods. Here, we describe a detailed protocol for both RNA FISH flow cytometry and confocal microscopy.

## [Background]

The barriers to development of antiviral drug resistance vary greatly depending on the compound used and the host or viral target chosen. RNA viruses have particularly low genetic barriers to the development of drug resistance as their polymerases have error rates as high as 10^−4^ to 10^−5^ misincorporations per nucleotide synthesized. This leads to exceptionally high genetic variability amongst progeny. However, the high level of diversity observed in RNA virus progeny does not always lead to high rates of genetic selection for progeny with increased fitness. This is often due to genetic dominance of drug-susceptible viruses that are present in the same cell as newly synthesized drug-resistant variants. Drug-resistant viral RNA must first be amplified and translated in its cell of origin, making newly synthesized drug-resistant viruses susceptible to dominant suppression by their drug-susceptible parents and cousins. We have coined the term “dominant drug targets” to describe viral targets with higher barriers to the development of antiviral drug resistance due to genetic dominance of drug-susceptible viruses. Study of genetic interactions and physical location of distinct viral genomes in the same cell required the development of the new technology described here.

To identify dominant drug targets for which tool antiviral compounds are available, we first generated drug-resistant viruses and built the mutations into an infectious cDNA clone. To test whether drug-resistant or drug-susceptible viruses were genetically dominant, we generated cells co-infected with drug-susceptible and drug-resistant viruses and then monitored selection from within them. In studies using poliovirus (Crowder and Kirkegaard, 2005; Tanner et al., 2014) and Dengue virus (Mateo et al., 2015) we were able to generate sufficiently high-titer virus stocks to perform coinfections at high multiplicities of infection and thus ensure that all cells in our cultures were coinfected. Recently, we expanded tests for dominance to hepatitis C virus (HCV) (van Buuren et al., 2018), for which high-titer stocks are often difficult to obtain, especially for drug-resistant variants that have reduced fitness. Therefore, when we co-infected Huh7.5.1 cells with two strains of HCV at multiplicities of infection of less than 1 PFU/cell, we generated four cell populations: co-infected cells, two types of singly infected cells and a significant population of uninfected cells. We needed to differentiate co-infected cells from the two types of singly infected cells and learn about genetic selection while doing so. To accomplish this, we were early adopters of the branched DNA (bDNA) technology originally developed by Affymetrix (now Thermo Fisher Scientific). This technology uses tiered DNA oligos to build a network of up to 8,000 fluorophores on each target RNA. This unique type of RNA fluorescent *in situ* hybridization (FISH) can be coupled with protein detection using standard antibody conjugation and detected using confocal microscopy (ViewRNA® Cell Plus Assay) and flow cytometry (PrimeFlow™ RNA Assay).

These bDNA FISH techniques first generate a series of target probes that bind the RNA of interest at adjacent sequences, but leave 3’ extensions of unique sequence to bind the pre-amplifier DNA that is complementary to two different probes. Cooperative binding of the pre-amplifier DNA to two probes increases the signal-to-noise ratio because any individual mistargeted probe cannot be amplified. Typically, twenty pairs of target probes are designed to bind the RNA of interest; this requires roughly 1,000 nucleotides of sequence space. Each of the twenty pre-amplifier DNAs is then bound by a series of amplifier DNAs, and then subsequently by a series of oligonucleotide-conjugated fluorophores. This process leads to the labeling of each individual target RNA with up to 8,000 fluorophores, sufficient to visualize individual RNAs by confocal microscopy. The PrimeFlow RNA Assay and ViewRNA Cell Plus Assay kits allow for simultaneous detection of three target RNAs. The available fluorophores for PrimeFlow are Alexa Fluor® 488, Alexa Fluor® 647 and Alexa Fluor® 750 and for ViewRNA are Alexa Fluor® 488, Alexa Fluor® 546 and Alexa Fluor® 647.

To apply this technology to dominant drug targeting in HCV, we needed to generate a strain of HCV with sufficient dissimilarity in its RNA sequence that we could differentiate it from wild-type viral RNA. To accomplish this, we generated three codon-altered versions of the JFH1 strain of HCV. Codon optimization algorithms available through GeneArt (Thermo Fisher Scientific) were used to design three approximately 1,000-nucleotide regions of the JFH1 genome that had altered codon usage but retained the same protein sequence. These codon-altered JFH1 strains all contained 200-250 synonymous mutations over the 1,000-nucleotide regions. Of these three strains, two demonstrated decreased fitness, likely due to disruption of RNA secondary structures required for viral replication (Pirakitikulr et al., 2016). The third strain, however, displayed growth kinetics that mimicked wild-type virus and could be used in co-infection experiments and differentiated from wild-type JFH1 using both RNA FISH and flow cytometry.

## Materials and Reagents

1. Pipette tips (with or without filter tips)
2. Micro slides (VWR, catalog number: 48311-702)
3. Micro cover glass (VWR, catalog number: 48380-046)
4. GenePulser cuvettes, 4 mm (Bio-Rad Laboratories, catalog number: 1652088)
5. BD FACS tubes (BD Falcon, catalog number: 352054)
6. 12-well cell culture dish (*e.g.*, Corning, Costar, catalog number: 3513)
7. 10 cm tissue culture dish (*e.g.*, Corning, catalog number: 430167)
8. T150 tissue culture flask (*e.g.*, Corning, catalog number: 430825)
9. 15 ml conical centrifuge tube (*e.g.*, AccuFlow, catalog number: EK-4020)
10. 500 ml Rapid-Flow Filter Unit, 0.2 μm (Thermo Fisher Scientific, catalog number: 566-0020)
11. Huh7.5.1 cells (Gift from Dr. Michael Gale Jr., University of Washington)
12. PrimeFlow™ RNA Assay Kit (Thermo Fisher Scientific, catalog number: 88-18005-210) contains:
  - Flow Cytometry Staining Buffer
  - Fixation Buffer 1
  - Permeabilization Buffer with RNase Inhibitors
  - Fixation Buffer 2
  - Wash Buffer
  - Target Probe Diluent
  - PreAmp Mix
  - Amp Mix
  - Label Probe Diluent
  - 100x Label Probes
13. ViewRNA® Cell Plus Assay Kit (Thermo Fisher Scientific, catalog number: 88-19000) contains:
  - Fixation/Permeabilization Buffer
  - Blocking/Antibody Diluent
  - Fixative
  - Probe Set Diluent
  - Amplifier Diluent along with Pre-Amplifiers and Amplifiers
  - Label Probe Diluent and Label Probes
  - Wash Buffer
  - PBS
  - DAPI
14. Target Probes (Thermo Fisher Scientific)
  - Wild-type JFH1 (VF1-14301)
  - Codon altered JFH1 (VF4-6000723)
15. Permafluor Mounting Reagent (Thermo Fisher Scientific, catalog number: TA-030-FM)
16. 0.05% Trypsin-EDTA (Thermo Fisher Scientific, Gibco, catalog number: 25300-054)
17. Xbal and CutSmart Buffer (New England Biolabs, catalog number: R0145L)
18. MEGAscript T7 Kit (Thermo Fisher Scientific, Invitrogen, catalog number: AMB1334-5)
19. Trizol® Reagent (Thermo Fisher Scientific, Ambion, catalog number: 15596018)
20. QIAquick PCR Purification Kit (QIAGEN, catalog number: 28106)
21. Human AB Serum (Omega, catalog number: HS-20)
22. Pen/Strep (Thermo Fisher Scientific, catalog number: 15140-122)
23. Glutamax (Thermo Fisher Scientific, catalog number: 35050-061)
24. Non-essential amino acids (Thermo Fisher Scientific, catalog number: 11140-050)
25. DMEM (GE Healthcare, Hyclone, catalog number: SH30243.01)
26. Fetal bovine serum (Omega, catalog number: FB-22)
27. KCl
28. CaCl_2_
29. K_2_HPO_4_
30. HEPES
31. EDTA
32. MgCl_2_
33. Human serum media (see Recipes)
34. 10% FBS media (see Recipes)
35. CytoMix (see Recipes)

## Equipment

1. Pipettes (with or without filter tips)
2. Ultrafine forceps (*e.g.*, Excelta, catalog number: 5-SN)
3. Modified BD FACScan (Scanford) or LSRII Flow Cytometer
4. Bio-Rad GenePulser XCell
5. Biosafety Cabinet (BSC)
6. Incubator (VWR, model: Model 1565)
7. Heat Block (*e.g.*, Anodized Aluminum, see Figure 2)
8. Leica SP8 Confocal Microscope (Leica Microsystems, model: Leica TCS SP8)
9. Sorvall Centrifuge (*e.g.*, Thermo Fisher Scientific, model: Legend RT plus)
10. Heracell 150i CO_2_ Incubator (Thermo Fisher Scientific, model: Heracell™ 150i)
−20 °C Freezer
12. Refrigerator

## Software

1. FlowJo® v10.0
2. Volocity v6.0 (PerkinElmer)
3. Adobe Photoshop vCS4
4. GraphPad Prism v7.0
5. Microsoft Excel v16.0

## Procedure

### A. Construction of codon altered sequences

**Figure 1.**
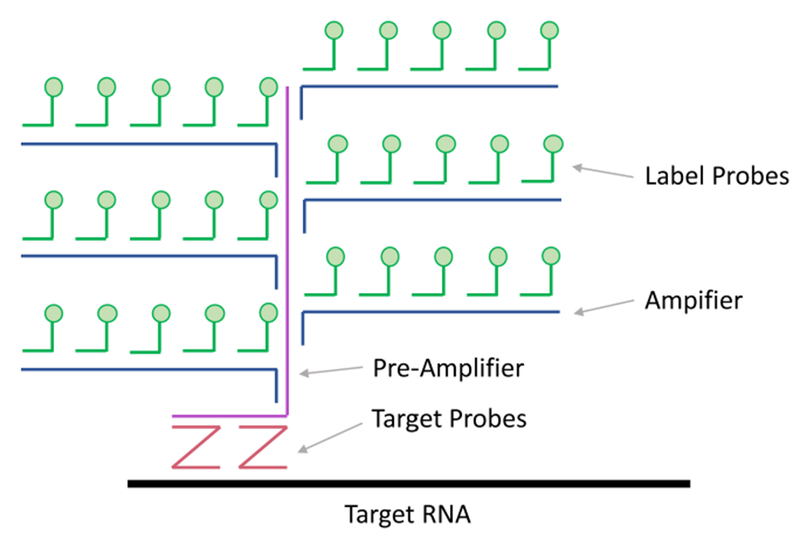
Detection of target RNAs using branched DNA technology. Branched DNA technology for RNA detection can be coupled with confocal microscopy or flow cytometry. Target RNAs are first bound by pairs of Target Probes. Typically, twenty sets of target probe pairs are designed per target RNA. The Pre-Amplifier DNA only binds target probe pairs that are bound to target RNAs in the correct orientation; this greatly limits the signal to noise ratio. Pre-Amplifier DNAs are then bound by Amplifier DNAs and subsequently by Label Probes. This process results in the labeling of target RNAs by up to 8,000 fluorophores.

1. Roughly 1,000 nucleotides of RNA sequence are required to support the hybridization of twenty bDNA trees and 8,000 fluorophores. Targets that contain less than the full complement of bDNAs can still be detected by flow cytometry but require higher copy numbers to achieve the same resolution.
2. For viral RNAs, when possible, scan the literature for any structural information available to determine which areas of the genome are the least likely to contain essential RNA secondary structures. If possible, also choose a region that has convenient cut sites for insertion of your codon-altered sequence. We chose to clone three codon-altered regions of the JFH1 genome because we anticipated decreased viability from some of the codon-altered strains.
3. GeneArt is a product offered through Thermo Fisher Scientific and can be used to synthesize genes up to 9,000 bp in length (https://www.thermofisher.com/us/en/home/life-science/cloning/gene-synthesis/geneart-gene-synthesis.html). The GeneArt homepage offers several tools, including the gene optimizer tool. Use the gene optimizer algorithms to design codon-altered sequences with wild-type viral RNA sequence as your template. We submitted three regions of JFH1 that were all roughly 1,000 nucleotides in length and flanked by convenient cut sites. The optimizer tool was able to alter nearly 25% of nucleotides in all three cases.
4. The newly synthesized sequence will arrive incorporated into a plasmid with a defined antibiotic-resistance marker. At this time, your codon-altered gene fragment can be subcloned into a plasmid that encodes the viral genome using restriction digestion and ligation.
5. Target Probes that differentiated viral RNA sequences were designed and manufactured by Affymetrix (now Thermo Fisher Scientific) for use with both the ViewRNA and PrimeFlow platforms (Figure 1).

### B. Collection of codon-altered JFH1 virus stocks

1. The pJFH1 plasmid encodes the full-length genome of the JFH1 strain of HCV. The wild-type plasmid and all codon-altered versions contain an Xbal cut site at the 3’ end of the genome. Digest 5 μg of plasmid DNA with 20 U of Xbal in the CutSmart Buffer provided in a final reaction volume of 25 μl. Incubate digestions at 37 °C for 2 h.
2. Purify linearized DNA using the QIAquick PCR Purification Kit, as per manufacturer’s protocol.
3. Using 1 μg of linearized plasmid as your template, perform *in vitro* transcription with the MEGAscriptT7 kit to make full-length genomic viral RNA. Incubate *in vitro* transcription reaction at 37 °C for 6 h. The temperature and duration of this incubation can be altered for optimal yield of individual transcripts.
4. Isolate synthesized viral RNA using Trizol as per the manufacturer’s protocol. Resuspend vRNA pellet in 50 μl of RNase-free water.
5. Seed 10^7^ Huh7.5.1 cells into a 10 cm tissue culture plate and incubate overnight.
6. To electroporate 10 μg vRNA into 10^7^ Huh7.5.1 cells to produce continuous HCV cultures:
  - Wash Huh7.5.1 cells with 5 ml PBS.
  - Add 2 ml of Trypsin and incubate at 37 °C for 5 min.
  - Add 5 ml of 10% FBS media and harvest cell suspension into a 15 ml conical tube.
  - Centrifuge cells at 400 *× g* for 4 min.
  - Resuspend cell pellet in 5 ml of PBS.
  - Centrifuge cells at 400 *× g* for 4 min.
  - Resuspend cell pellet with 5 ml of CytoMix. Cytomix recipe can be found below under “Recipes”.
  - Centrifuge cells at 400 *× g* for 4 min.
  - Resuspend cell pellet in 400 μl of CytoMix and transfer to a 4 mm GenePulser cuvette.
  - Mix 10 μg of viral RNA into cell suspension inside cuvette and gently pipet up and down to mix.
  - Electroporate RNA-cell mixture using the Bio-Rad GenePulser XCell. Settings set to 950 μF capacitance, 270 V, ∞ resistance and 4 mm cuvette size.
  - Allow cells to rest at room temperature for 10 min.
  - Transfer electroporated cells to a fresh 10 cm culture dish with 10 ml of 10% FBS media (see Recipes).
7. Culture electroporated cells for up to two weeks in 10% FBS media, passaging every 3-4 days as required. As you passage, expand the culture. Typically, cultures of 10^7^ electroporated cells are expanded into either five or ten T150 flasks. This gives HCV time to spread and generates a culture with a higher percentage of cells that are infected and productively synthesizing progeny virus. Further expansion of cells to larger capacity can be done if needed.
8. Convert JFH1 cultures to Human Serum Media (Steenbergen et al., 2013). Growth of HCV in human serum has two benefits. First, Huh7.5.1 cells differentiate and cease cell division, therefore trypsinization and biweekly passage are no longer required. Instead, virus containing cell supernatants can simply be collected biweekly and directly replaced with fresh medium. Second, growth in Human Serum Media increases viral yield by 10 to 100-fold.

### C. Simultaneous infection with two HCV strains and detection of co-infected cells with Prime-Flow.

**Figure 2.**
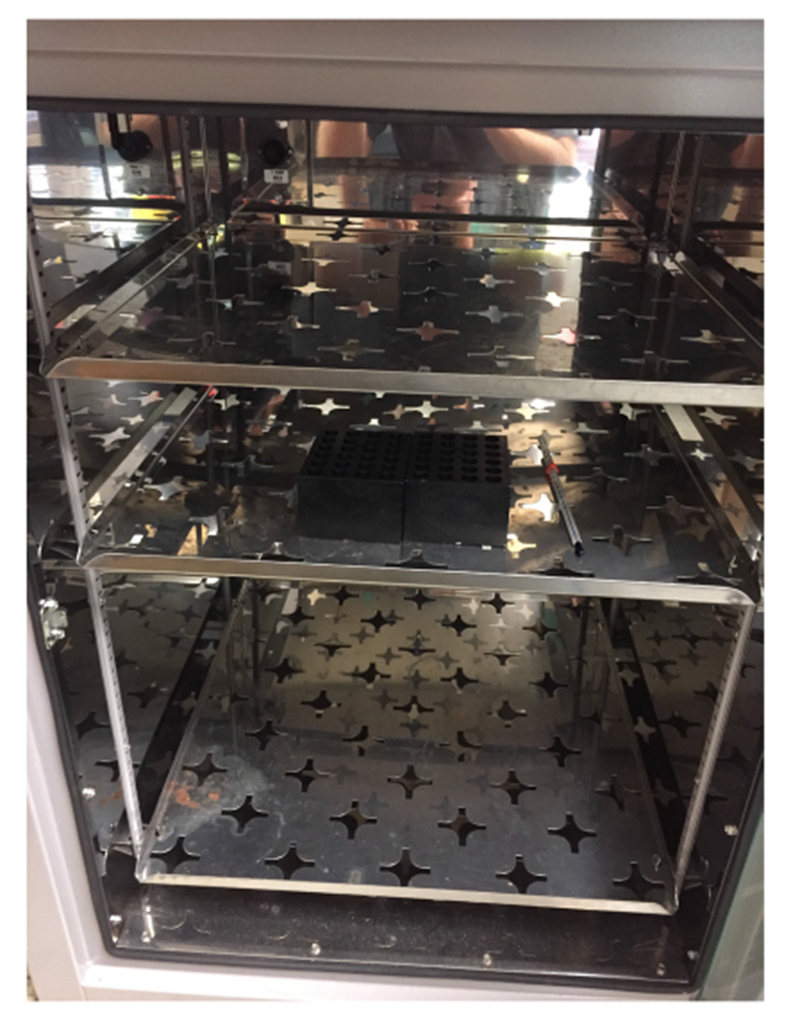
40 ± 1 °C incubator setup. Two heat blocks are stored in the incubator to regulate the temperature of RNA FISH flow cytometry samples. A thermometer is kept inside to confirm the digital temperature readings.

1. The description of this protocol has been adapted from the PrimeFlow Assay user’s manual.
2. Huh7.5.1 cells are seeded into 12-well plates at a density of 10^5^ cells per well using 1 ml of 10% FBS media.
3. In our hands, JFH1 cultured in human serum media can produce viral titers of 10^5^-10^6^ focus forming units (FFU) per ml. Infect Huh7.5.1 cells at a multiplicity of infection of one virus particle per cell with both wild-type and codon-altered JFH1. This often equates to roughly 1-2 ml of each virus preparation. A total volume of 4 ml can be used carefully in 12-well plates.
4. Incubate infected cells in a CO_2_ incubator at 37 °C for 4-6 h. Following initial incubation, remove virus-containing media by aspiration. Replace media with fresh 10% FBS media and incubate infected cells for 72 h.
5. Replace 10% FBS media with fresh 10% FBS media that either contains antiviral drugs or vehicle and incubate infected cells for 24-36 h.
6. Aspirate off media containing antivirals or vehicle and wash cells with 1 ml PBS.
7. Harvest infected cells by treating cells with 0.5 ml trypsin and incubating at 37 °C with CO_2_ for 5 min.
8. Inhibit trypsin by adding 1 ml of 10% FBS media to each well. Harvest all cells and transfer to one of the 1.5 ml microfuge tubes supplied in the PrimeFlow Assay kit.
9. Spin cells at 400 *× g* for 5 min.
10. Aspirate off media and trypsin, being careful not to lose any cells. This is achieved by only aspirating down to the 100 μl marker on the side of the Eppendorf tube. Wash cells with 1 ml of Flow Cytometry Staining Buffer. Vortex and spin at 400 *× g* for 5 min.
11. Aspirate Flow Cytometry Staining Buffer and fix cells using 1 ml of Fixation Buffer 1 at 4 °C for 30 min.
12. Spin cells at 800 *× g* for 5 min.
13. Resuspend cells in 1 ml of Permeabilization Buffer. Spin cells at 800 *× g* for 5 min. Repeat wash with Permeabilization Buffer 3 ×.
14. Aspirate final Permeabilization Buffer wash and resuspend cells in 1 ml of Fixation Buffer 2. Incubate cells in the dark at room temperature for 60 min.
15. Spin cells at 800 *× g* for 5 min and resuspend in 1 ml of Wash Buffer.
16. Repeat wash step.
17. Dilute Target Probes in Target Probe Diluent at 1:20.
18. Resuspend cells in 100 μl of the Target Probe mixture. Incubate at 40 ± 1 °C for 2 h. We use a heat block in our 40 °C incubator to increase heat conduction to the tubes and protect from large fluctuation in heat (Figure 2). This incubation can be extended from 2 h to overnight. Longer incubations periods allowed for all amplification steps, flow cytometry and data analysis to be completed the following day.
19. Wash cells by adding 1 ml of Wash Buffer, vortex, and spin at 800 *× g* for 5 min.
20. Repeat wash step.
21. Resuspend cells in 100 μl of PreAmp Mix. Incubate at 40 ± 1 °C for 1.5 h.
22. Wash cells by adding 1 ml of Wash Buffer, vortex, and spin at 800 *× g* for 5 min.
23. Repeat wash step.
24. Resuspend cells in 100 μl of Amp Mix. Incubate at 40 ± 1 °C for 1.5 h.
25. Wash cells by adding 1 ml of Wash Buffer, vortex, and spin at 800 *× g* for 5 min.
26. Repeat wash step.
27. Prepare Label Probe mix by diluting Label Probes into the Label Probe Diluent at 1:100.
28. Resuspend cells in 100 μl of Label Probe mix. Incubate at 40 ± 1 °C for 1 h.
29. Wash cells by adding 1 ml of Wash Buffer, vortex, and spin at 800 *× g* for 5 min.
30. Repeat wash step.
31. Aspirate Wash Buffer leaving 100 μl of residual liquid to resuspend stained cells. Resuspend cells by pipetting up and down and transfer to a labeled BD FACS tube containing 250 μl of PBS.
32. Analyze cells using a flow cytometer and FlowJo software (details below).

### D. Quantitation of RNA-protein colocalization using confocal microscopy.

**Figure 3.**
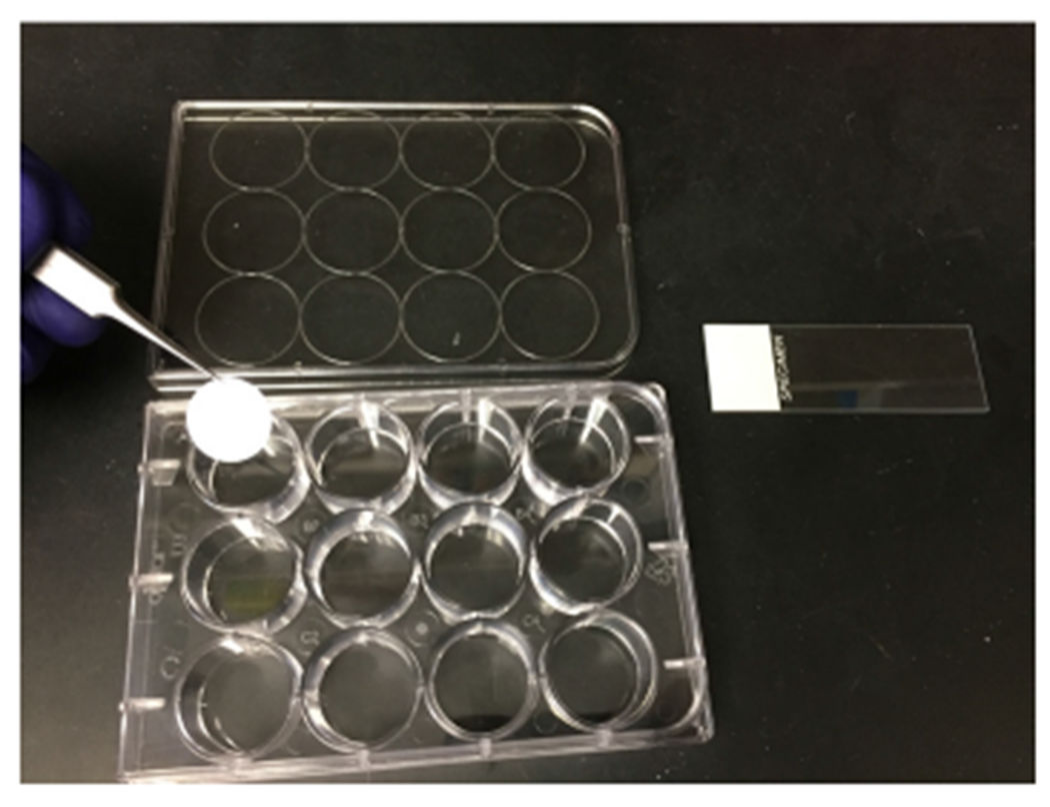
Reagents setup for confocal microscopy. Huh7.5.1 cells are plated onto Micro Cover Glass within a 12-well tissue culture plate. These cells are infected following a 24 h incubation to allow cell adherence to the glass. Following infection, the cells are fixed, stained for protein and RNA using the ViewRNA Cell Plus Assay kit, all within the 12-well plate. The Micro Cover Glass is then carefully transferred to a Microslide spotted with PermaFluor/DAPI using fine forceps.

1. Huh7.5.1 cells are plated on Micro Cover Glass inside 12-well tissue culture plates at a density of 10^5^ cells per well one day prior to infection (Figure 3).
2. Co-infect cells with wild type JFH1 and codon altered JFH1 at a multiplicity of infection equal to one virus per cell.
3. At 6 h post infection, aspirate inoculum and replace with 1 ml of 10% FBS media.
4. At 24 h post infection, wash cells 2 × with 1 ml of PBS.
5. Add 400 μl of Fixation/Permeabilization Buffer to each well and incubate for 30 min at room temperature.
6. Wash cells 3 × each with 800 μl of PBS.
7. Overlay cells with 400 μl of Blocking/Antibody Diluent and incubate at room temperature for 20 min.
8. Dilute primary antibody in 400 μl Blocking/Antibody Diluent as required. Overlay cells with antibody mixture and incubate at room temperature for 1 h.
9. Wash cells three times with PBS.
10. Dilute secondary antibody in 400 μl Blocking/Antibody Diluent as required. We use anti-mouse AlexaFluor-647 diluted at 1:200 for our experiment with HCV. Overlay cells with antibody mixture and incubate at room temperature for 1 h.
11. Wash cells 3 × with PBS.
12. Add 400 μl of Fixation Solution to each well and incubate in the dark at room temperature for 1 h.
13. Wash cells 3 × with PBS.
14. Dilute Target Probes1:100 in Target Probe Diluent.
15. After the final wash, overlay cells with 400 μl of Target Probe mixture. Incubate at 40 ± 1 °C for 2 h.
16. Wash cells 3 × with 800 μl Wash Buffer at room temperature.
17. Dilute Pre-Amplifiers 1:25 in Amplifier Diluent.
18. After the final wash, overlay cells with 400 μl of Pre-Amplifier mixture and incubate at 40 ± 1 °C for 1 h.
19. Wash cells 3 × with 800 μl Wash Buffer at room temperature.
20. Dilute Amplifiers 1:25 in Amplifier Diluent.
21. After the final wash, overlay cells with 400 μl of Amplifier mixture and incubate at 40 ± 1 °C for 1 h.
22. Wash cells 3 × with 800 μl Wash Buffer at room temperature.
23. Dilute Label Probes 1:100 in Label Probe Diluent.
24. After the final wash, overlay cells with 400 μl of Label Probe mixture and incubate at 40 ± 1 °C for 1 h.
25. Wash cells 3 × with 800 μl Wash Buffer at room temperature.
26. Dilute DAPI 1:100 in Permafluor mounting reagent.
27. Spot 12.5 μl of Permafluor/DAPI mixture onto a Micro Slide.
28. Using forceps, carefully remove stained Micro Cover Glass from the 12-well dish, dab on a Kimwipe to remove excess Wash Buffer and place “cells down” onto the drop of Permafluor/DAPI. Allow to harden for at least 4 h.
29. Visualize cells using a confocal microscope. We use a Leica SP8 Confocal Microscope fitted with a White Light Laser.

## Data analysis

### Flow cytometry

**Figure 4.**
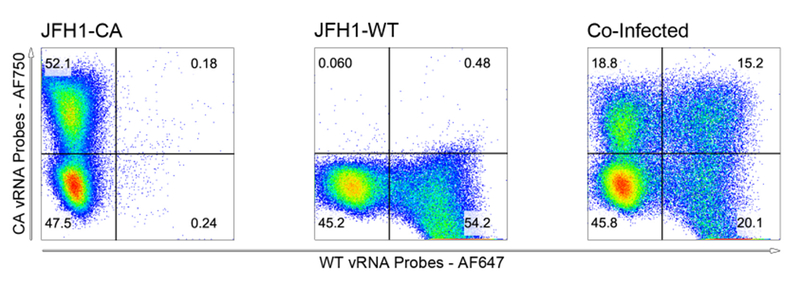
Identification of co-infected cells by PrimeFlow RNA FISH. Huh7.5.1 cells were infected with JFH1-CA, JFH1-WT or co-infected at multiplicities of infection equal to one virus per cell with each virus. Infected cells were incubated for 72 h before labeling viral RNAs using PrimeFlow. Analysis and compensation was performed using FlowJo.

We analyze all flow cytometry data using FlowJo software. Data are exported from the flow cytometer as individual .fcs files for each sample as well as a .wsp file for the entire experiment. We use FlowJo to open the .wsp file and can then access all .fcs files in the same analysis window. Once files are open in FlowJo data analysis proceeds as follows:

1. Open your first sample and select forward scatter versus side scatter to view cells collected. Draw a gate around the healthy cells only so that any debris or dead cells are not included in your analysis.
2. Within your healthy cell subgate, plot the two viral RNA fluorophores against one another. In our case this was typically Alexa Fluor 488 versus Alexa Fluor 750 which did not require compensation. If you are working with Alexa Fluor 657 and Alexa Fluor 750 you will need to run the compensation algorithm within FlowJo before further analysis.
3. Once data are plotted and compensated, if needed, reset the axes to biexponential (Biex) which minimizes the uninfected cells and emphasizes the viral RNA-positive populations for clearer resolution.
4. Draw quadrants that divide uninfected cells from the two singly infected cells and coinfected cells.
5. The percentages from each population will be used to determine the dominance relationships between viral species. In the absence of drug, four cell populations will be visible. In the presence of drug, the cells singly infected with drug-susceptible virus will become uninfected and shift into the lower left quadrant. The singly infected drug-resistant virus will persist. The genetic outcome of the co-infected cells will determine their fate (Figure 4).

### Confocal microscopy

**Figure 5.**
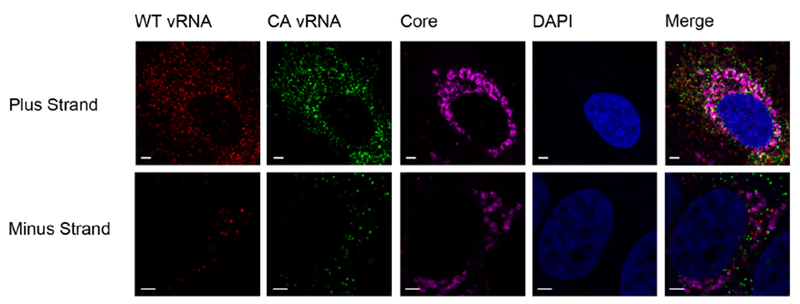
Analysis of viral RNA-protein colocalization using ViewRNA Cell Plus. Huh7.5.1 cells were co-infected with JFH1-WT and JFH1-CA on Micro Cover Glass for 72 h. Cells were stained for HCV core protein and both viral RNAs using the ViewRNA Cell Plus Assay. Quantification of colocalization was performed using Volocity software. Scale bars are 2.5 μm in length.

1. The Leica SP8 creates a file containing all images as a .lif file. The individual channels are exported as individual .tif files for image processing and figure construction (Figure 5).
2. The .lif file can also be opened using Volocity software created by PerkinElmer.
3. Volocity has a spot-counting algorithm to determine how many puncta exist within each channel. We define a single punctum as larger than 0.1 μm^2^ and smaller than 0.25 μm^2^, and ask Volocity to break larger spots into individual units. Confirm that your size range is appropriate by giving a few cells an eye test. Does the number of puncta counted appear to be the same number that you can count by eye? You may need to adjust your maximum and minimum punctum sizes based on this test.
4. We then ask Volocity to determine colocalization by counting how many spots on our Red channel shared at least 0.05 μm^2^ of “Mutual Space” with puncta the Green channel. The result is plotted as the total number of puncta that share mutual space between channels versus the total number of puncta in each channel.
5. Determining colocalization between RNA and protein requires a separate algorithm as the proteins often not localize into discrete countable puncta. We therefore ask Volocity to determine how many of the viral RNA puncta “Touch” anywhere within the protein signal. We then graph the number of RNA puncta that touched protein versus the total number of RNA puncta in each cell.

## Notes

1. We prefer to use the swinging bucket Sorvall centrifuge for all spins for flow cytometry as the cell pellet accumulates at the bottom of the PrimeFlow assay kit-supplied microfuge tubes, which limits cell loss during the multiple-step procedure. However, it is possible to complete the protocol and limit cell loss using a traditional bench top, fixed-angle centrifuge, with careful supernatant removal.
2. Simultaneous analysis of the Alexa Fluor 647 and Alexa Fluor 750 channels requires a high degree of compensation. To identify double-positive cells unambiguously, use Alexa Fluor 488 in combination with either of the other two channels.
3. As PrimeFlow is often coupled with antibody staining, it should be noted that not all fluorophores survive the RNA staining protocol. Specifically, all PerCP fluorophores will be inactivated by this technique and should be avoided in panel design.
4. Coverglass slips are very delicate and can easily break as they are being lifted out of the 12-well plate and placed onto the microslide. Using anything other than fine forceps makes this challenging. New students in our lab are encouraged to practice this technique using blank Micro Cover Glass in PBS prior to attempting a real experiment.

## Recipes

1. Human serum media
  2% human AB serum
  1× Pen/Strep
  1× glutamax
  1× non-essential amino acids
  DMEM
2. 10% FBS media
  10% fetal bovine serum
  1× Pen/Strep
  1× glutamax
  1× non-essential amino acids
  DMEM
3. CytoMix
  120 mM KCl
  0.15 mM CaCl_2_
  10 mM K_2_HPO_4_
  25 mM HEPES
  2 mM EDTA
  5 mM MgCl_2_
  Adjust pH to 7.6
  Filter through a 0.2 μm Rapid-Flow Filter Unit
